# Impact of maternity care policy in Catalonia: a retrospective cross-sectional study of service delivery in public and private hospitals

**DOI:** 10.1186/s12884-015-0446-y

**Published:** 2015-02-13

**Authors:** Ramón Escuriet-Peiró, Josefina Goberna-Tricas, Maria J Pueyo-Sanchez, Neus Garriga-Comas, Immaculada Úbeda-Bonet, Carmen Caja-López, Isabel Espiga-López, Vicente Ortún-Rubio

**Affiliations:** Department of Experimental and Health Sciences, Universitat Pompeu Fabra (UPF), Barcelona, Spain; Directorate-General for Health Planning and Research, Ministry of Health of the Government of Catalonia, Barcelona, Spain; Department of Public Health, Mental Health and Perinatal Nursing, Universitat de Barcelona, Bellvitge Health Sciences Campus, Pavelló de Govern, 3a planta, C/Feixa Llarga s/n, 08907 L’Hospitalet de Llobregat Barcelona, Spain; Hospital de Manresa, Fundació Althaia, Manresa, Spain; Observatory on Women’s Health, Subdirectorate for Quality and Cohesion, Ministry of Health, Social Services and Equality, Madrid, Spain; Faculty of Economic and Business Sciences, Universitat Pompeu Fabra (UPF), Barcelona, Spain; Department of Public Health, Mental Health and Perinatal Nursing. Bellvitge Health Sciences Campus, Universitat de Barcelona, Despatx 321 Pavelló de Govern, 3a planta, C/ Feixa Llarga s/n, 08907 L’Hospitalet de Llobregat, Barcelona, Spain

**Keywords:** Obstetric interventions, Birth, Maternity care

## Abstract

**Background:**

As a result of the growing number of interventions that are now performed in the context of maternity care, health authorities have begun to examine the possible repercussions for service provision and for maternal and neonatal health. In Spain the *Strategy Paper on Normal Childbirth* was published in 2008, and since then the authorities in Catalonia have sought to implement its recommendations. This paper reviews the current provision of maternity care in Catalonia.

**Methods:**

This was a descriptive study. Hospitals were grouped according to their source of funding (public or private) and were stratified (across four strata) on the basis of the annual number of births recorded within their respective maternity service. Data regarding the distribution of obstetric professionals were taken from an official government survey of hospitals published in 2010. The data on obstetric interventions (caesarean, use of forceps, vacuum or non-specified instruments) performed in 2007, 2010 and 2012 were obtained by consulting discharge records of 44 public and 20 private hospitals, which together provide care in 98% of all births in Catalonia. Proportions and confidence intervals were calculated for each intervention performed in all full-term (37–42 weeks) singleton births.

**Results:**

Analysis of staff profiles according to the stratification of hospitals showed that almost all the hospitals had more obstetricians than midwives among their maternity care staff. Public hospitals performed fewer caesareans [range between 19.20% (CI 18.84-19.55) and 28.14% (CI 27.73-28.54)] than did private hospitals [range between 32.21% (CI 31.78-32.63) and 39.43% (CI 38.98-39.87)]. The use of forceps has decreased in public hospitals. The use of a vacuum extractor has increased and is more common in private hospitals.

**Conclusions:**

Caesarean section is the most common obstetric intervention performed during full-term singleton births in Catalonia. The observed trend is stable in the group of public hospitals, but shows signs of a rise among private institutions. The number of caesareans performed in accredited public hospitals covers a limited range with a stable trend. Among public hospitals the highest rate of caesareans is found in non-accredited hospitals with a lower annual number of births.

## Background

Childbirth is one of the most common reasons for hospital admission in Spain [1]. One of the key responsibilities of health policymakers is to plan adequate maternity services and to provide the resources needed to ensure that care is both safe and of high quality.

Recent decades have seen an increasing medicalization of maternity care as a whole, most notably during labour, where various interventions may now be performed. [2] For some sectors of society, such developments are regarded as only to be expected and as a sign of progress. However, the observed outcomes in terms of health are beginning to be viewed with concern, since exposure to unjustified or unnecessary interventions may increase the risk of avoidable harm being caused to both mother and child [3-5]. In addition, our government is increasingly examining the economic costs and repercussions for health services of a non-rational use of resources [6].

Some research in this field has suggested that it would be helpful to establish a set of agreed criteria of ‘normality’, such that women who met these criteria could then receive maternity care in a setting that was less technologized and more geared towards normal childbirth, which could even be set apart from the conventional obstetric department. [7] Another topic of debate concerns the model of care provided. Some authors argue in favour of more person-centred care with a focus on the needs expressed by the pregnant woman [8,9] However, such concepts are not always applied or interpreted in the same way [10-12], and what is actually implemented may therefore differ across healthcare providers. Nevertheless, in recent decades women, as end users of these services, have become key protagonists when it comes to deciding the kind of maternity care they want, and they have called for greater respect to be shown towards their wishes; in this context, user groups have sometimes put considerable pressure on health policymakers to ensure that the care offered is more respectful of the physiology of labour [13].

In 2008, Spain’s Ministry of Health, Social Policy and Equality published the *Strategy for Assistance at Normal Childbirth in the National Health System*, which marked a change of direction in the maternity care offered within the public health service [14]. Publication of this strategy paper was followed by a series of actions to promote maternity services which were more clearly centred on the woman’s needs and were based on the concept of childbirth as a normal physiological process in which intervention was only required if problems were detected. In Catalonia, in the north-east of Spain the health authorities responded to the strategy paper by setting up a project designed to implement its recommendations in public hospitals.

Currently, the national health system in Catalonia comprises 44 public owned or state assisted hospitals and 43 reproductive health care units in the community. Antenatal and postnatal care for women not at risk is mainly given at these units by midwives, and delivery care is performed in hospitals staffed by teams of midwives and obstetricians. All women have access to these public services from the beginning of their pregnancy. Women who opt for private care take out private health insurance or contact professionals directly. Since the beginning of the project, the Department of Health has encouraged public hospitals to join; they are required to meet a number of conditions and undertake to implement the recommendations.

This project established three priority goals:

- Accreditation of hospitals, which would receive extra funding in order to adapt infrastructure within their maternity services;
- Training and awareness-raising for professionals;
- Involving women in decisions about their labour and treatment.

The requirements that the hospitals had to comply with included: establishing a system of coordination with the community care services, developing protocols for normal birth care, promoting the participation of women in decision-making and undertaking to adapt their infrastructure and provide space to care for women at low obstetric risk.

A series of workshops, sessions and courses on specific areas of childbirth care were held in order to train professional staff. To promote the participation of women, a “birth plan” was introduced.

Under the public health system, maternity care is available to all women living in Catalonia. This service includes provision of antenatal and postnatal care at community health centers and delivery care in maternity hospitals. Broadly speaking, midwives care for low-risk women throughout the process, and obstetricians take charge in the case of risk. Some women opt for private care; in such cases, care is provided by an obstetrician and the midwife works with the obstetrician during delivery care.

As several years have passed since this project was first implemented a process of evaluation is now underway, the aim of which is to assess the impact that the health policy set out in the 2008 strategy paper has had on maternity services in Catalonia. The evaluation process includes visits to accredited hospitals to determine the extent to which current practices promote a more woman-centred approach. In these visits we record information on the use of “birth plan”, continuity of care and the initiatives introduced to encourage participation and decision-making among women regarding the care they wish to receive during childbirth. We also analyse a series of indicators chosen to provide information about treatment practices within maternity services. These indicators examine aspects such as the use of obstetric interventions that are regarded as incompatible with normal childbirth (e.g. caesareans, the use of forceps, vacuum or unspecified instruments), as well as the kind of professional who takes the lead in the case of low-risk births. The category “unspecified instruments” includes the spatula, an obstetric instrument comprising two independent, non-articulated blades which adapt to the head of the fetus and which, unlike the forceps, act by pulsion rather than by traction. This type of instrument does not have a specific coding and so it is described here as “unspecified”.

This paper presents the results from a part of this evaluation process, and includes information relating to both public and private hospitals. The specific objectives of this research were:

- To identify trends in the kind of obstetric interventions performed (caesarean, use of forceps, vacuum extractor or spatulas classified as non-specified instruments), taking as a reference the year prior to publication of the strategy paper on normal childbirth (i.e. 2007) and comparing the data with those for 2010 and 2012, two and four years after its recommendations were first implemented in Catalonia;
- To determine the distribution of obstetric professionals (i.e., obstetricians and midwives) who work in public and private hospitals in Catalonia and their terms of employment with their respective hospitals.

## Methods

This was a descriptive study that aimed to examine changes in a series of indicators across three time points (2007, 2010 and 2012). The indicators considered concerned the use of caesarean section, forceps, a vacuum extractor or non-specified instruments during full-term (37–42 weeks) singleton births in Catalonia. These data were obtained by consulting the hospital discharge register, the Minimum Basic Data Set (MBDS). The register is mandatory for all public hospitals and is the basis for reimbursement. Each hospital discharge is registered with administrative information on the patient, hospital episode and hospital. The diagnoses are coded according to the International Classification of Diseases, Ninth Revision, Clinical Modification (ICD-9-CM). Information is included from forty-four state assisted hospitals offering public services (public hospitals) and 20 of the region’s 27 private hospitals.

In line with the second study objective, this paper also presents descriptive data regarding the distribution of obstetric staff in the two groups of hospitals. This information was extracted from an official government survey of hospitals that was published in 2010.

For the purposes of analysis, hospitals were classified as either public or private, and they were stratified (across four strata) according to the annual number of births recorded in their respective maternity service: S1: <600 births/year; S2: 600–1200 births/year; S3: 1201–2400 births/year; S4: >2400 births/year. Public hospitals were further classified according to whether or not they had been accredited to implement the recommendations of the 2008 strategy paper on normal childbirth. This classification (accredited vs. non-accredited) was made separately for the years 2010 and 2012. The unit of analysis in the present study is *the hospital*, it being assumed that this represents the overall effect of the organization on the likelihood of a given obstetric intervention being performed.

In order to observe any changes in the chosen indicators we took as a reference the year prior to publication of the strategy paper on normal childbirth (i.e. 2007) and compared the data with those for 2010 and 2012, two and four years after its recommendations began to be implemented in Catalonia. We first obtained an overview of any changes in the chosen indicators across the three time points. To do so, we examined the number of obstetric interventions performed at all hospitals. The aim here was to observe the trend for Catalonia as a whole across the study period.

A descriptive analysis was carried out for each group of hospitals. For each stratum we calculated proportions and confidence intervals (95%) for each indicator. We recorded the use (yes/no) of each obstetric intervention considered during full-term (37–42 weeks) singleton births. To determine whether the proportion of obstetric interventions had varied since the beginning of the project, a comparison of proportions was performed on the strata of the two groups of hospitals between 2007 and 2012 using the Z test (level of significance α = 0.05).

### Ethical approval

This study was exempt from review by the Ethics Committee of the Catalan Ministry of Health as it used publicly available, anonymised data. Furthermore, this paper forms part of the objectives set out in Project FEM2012-33067, *Maternity, Technology and Healthcare Relationships*”, which has received approval from the Bioethics Committee of the University of Barcelona.

## Results

**Figure 1:**
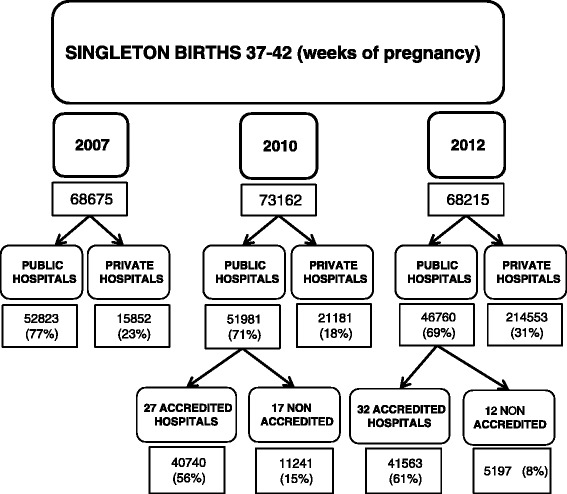
**Distribution of singleton births in public and private hospitals.**

This study includes all births attended during the years studied at 44 public hospitals and 20 private hospitals, representing 98% of all births attended in Catalonia. During the study period the majority of full-term singleton births in Catalonia took place within public hospitals, although the proportion fell from 77% in 2007 to 69% in 2012. In 2010 a total of 27 public hospitals had been accredited to implement the normal childbirth initiative, and they provided care in 78% of births in public hospitals. By 2012 a further 5 hospitals had been accredited, and together these 32 institutions provided care in 88% of all full-term singleton births in the group of public hospitals (Figure 1).

Table 1 gives detail of women’s age at the time of giving birth, the mean age of women who gave birth in public hospitals was lower at all three time points studied.

**Table 1:** **Singleton births average maternal age in public and private hospitals**

	**Average maternal age**	
**Year**	**Public hospitals**	**Private hospitals**
2007	29,89 (SD 5.47)	32.84 (SD 3.93)
2010	30,32 (SD 5,50)	33,38 (SD 3,90)
2012	30,75 (SD 5,60)	33,73 (SD 4,07)

### Obstetric professionals

All hospitals in Catalonia have more obstetricians than midwives. The staff’s employment situation depends on the type of hospital: public hospitals have a higher proportion of directly employed full-time or part-time staff, meaning that they are physically present at the hospital, whereas private hospitals have a higher proportion of associate health professionals, which generally means that they are not based at the hospital and only attend when required (i.e. “on call”). This pattern is observed for both obstetricians and midwives in both groups of hospitals. The greater number of obstetricians than midwives is found in all types of hospitals studied, regardless of whether they have more permanent or more associate staff, with just one exception: public hospitals classified as S3 (1201–2400 births/year) had more midwives than obstetricians (Table 2).

**Table 2:** **Health professional’s distribution in public and private hospitals**

		Hospital staff*		**Associate health professionals**	Hospital staff*		**Associate health professionals**	Hospital staff*		**Associate health professionals**	Hospital staff*		**Associate health professionals**
	**Total**	**Total**	**N (%)**	**N (%)**	**Total**	**N (%)**	**N (%)**	**Total**	**N (%)**	**N (%)**	**Total**	**N (%)**	**N (%)**
**Stratum**		**S1**			**S2**			**S3**			**S4**		
**Number of public hospitals**	**43**	**11**			**11**			**16**			**5**		
**Obstetricians**	**625**	**67**	66(98.50%)	1(1.49%)	**121**	119(98.34%)	2(1.65%)	**254**	254(100.00%)	0(0.00%)	**183**	181(98.90%)	2(1.09%)
**Mildwives**	**600**	**55**	52(5.45%)	3(5.45%)	**99**	98(98.98%)	1(1.01%)	**313**	307(98.08%)	6(1.91%)	**133**	133(100.00%)	0(0.00%)
**Number of private hospitals**	**16**	**5**			**3**			**3**			**5**		
**Obstetricians**	**493**	**94**	24(25.53%)	70(74.46%)	**52**	4(7.69%)	48(92.30%)	**77**	1(1.29%)	76(98.70%)	**270**	14(5.18%)	256(94.81%)
**Midwives**	**169**	**50**	28(56.00%)	22(44.00%)	**13**	1(7.69%)	12(92.30%)	**42**	5(11.62%)	38(88.37%)	**63**	25(39.68%)	38(60.31%)

Hospital Staff*. includes health professionals working Full Time and Part Time.

### Obstetric interventions

**Figure 2:**
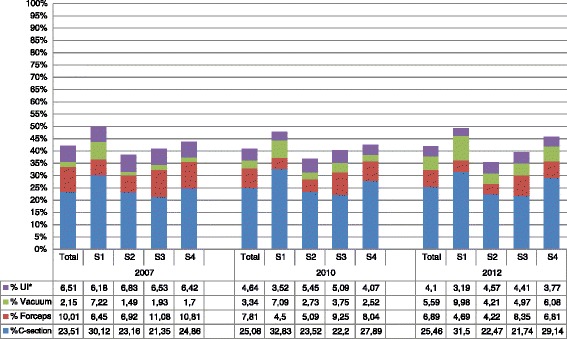
**Obstetric interventions in all hospitals by stratum.**

The most common procedures carried out at the hospitals were caesareans: the proportions for the other kinds of intervention considered varied across strata and by year (Figure 2).

Hospitals classified as S1 (lowest number of births/year) performed the highest mean number of caesareans at all three time points. These hospitals also performed the highest number of obstetric interventions overall in all three years considered. When each stratum is considered separately the data show that in hospitals classified as S4 (highest number of births/year) the number of caesareans performed has increased from 24.86% (CI 24.47-25.25) in 2007 to 29.14% (CI 28.73-29.56) in 2012. In terms of the use of forceps, this has progressively decreased in all strata. All four strata show a trend towards an increased use of a vacuum extractor and a decrease in the use of non-specified instruments.

Table 3 shows data (including proportions and the corresponding confidence interval) for the types of obstetric interventions performed in each group of hospitals, by year and by stratum. The most relevant findings are summarized in the following two-sub-sections.

**Table 3:** **Obstetric interventions in public and private hospitals by stratum**

		**C-Section**		**Forceps**		**Vacuum**		UI*	
		**%**	***CI***	**%**	***CI***	**%**	***CI***	**%**	***CI***
**Public hospitals**									
**2007**	**S1**	28.13	*26.36-29.91*	6.68	*5.69-7.66*	2.73	*2.08-3.37*	6.72	*5.73-7.74*
	**S2**	21.47	*20.65-22.30*	7.10	*6.59-7.62*	0.32	*0.20-0.43*	7.00	*6.48-7.51*
	**S3**	19.19	*18.71-19.69*	11.97	*11.56-12-37*	0.38	*0.30-0.46*	6.30	*5.99-6.60*
	**S4**	19.49	*18.89-20.11*	13.21	*12.69-13.73*	0.35	*0.26-0.44*	4.08	*3.77-4.38*
**2010**	**S1**	28.69	*27.05-30.33*	5.44	*4.61-6.26*	2.27	*1.73-2.81*	3.03	*2.40-3.65*
	**S2**	19.33	*18.50-20.15*	6.02	*5.53-6.52*	1.74	*1.46-2.01*	5.13	*4.67-5.59*
	**S3**	19.34	*18.85-19.83*	9.94	*9.57-10.32*	2.55	*2.36-2.75*	5.00	*4.73-5.27*
	**S4**	19.46	*18.83-20.09*	11.16	*10.66-11.66*	1.05	*0.89-1.21*	1.71	*1.50-1.92*
**2012**	**S1**	25.11	*23.19-27.04-*	6.48	*5.39-7.58*	2.52	*1.82-3.21*	2.16	*1.51-2.81*
	**S2**	19.78	*18.93-20.63*	4.77	*4.32-5.22*	2.99	*2.63-3.35*	4.34	*3.90-4.77*
	**S3**	19.63	*19.12-20.15*	8.98	*8.61-9.35*	3.67	*3.42-3.91*	4.35	*4.09-4.61*
	**S4**	21.44	*20.74-22.14*	9.70	*9.19-10.20*	2.00	*1.87-2.23*	1.15	*0.97-1.34*
**Private hospitals**									
**2007**	**S1**	32.21	*30.31-34.10*	6.21	*5.23-7.19*	11.95	*10.63-13.26*	5.61	*4.68-6.54*
	**S2**	35.66	*33.03-38.29*	5.56	*4.31-6.28*	10.19	*8.53-11.85*	5.56	*4.31-6.82*
	**S3**	36.10	*34.54-37.66*	5.04	*4.33-5.75*	12.48	*11.41-13,56*	8.10	*7.21-8.99*
	**S4**	34.93	*33.92-35.93*	6.28	*5.77-6.79*	4.23	*3.80-4,65*	10.82	*10.17-11.48*
**2010**	**S1**	38.65	*36.55-40.75*	3.19	*2.43-3.95*	13.86	*12.38-15.35*	4.20	*3.34-5.07*
	**S2**	38.15	*36.27-40.04*	1.85	*1.32-2.37*	6.21	*5.27-7.15*	6.56	*5.60-7.52*
	**S3**	38.33	*36.65-40.02*	3.79	*3.13-4.45*	13.12	*11.95-14.29*	6.05	*5.22-6.87*
	**S4**	37.49	*36.67-38.31*	4.50	*4.15-4.85*	4.20	*3.86-4.53*	6.75	*6.33-7.18*
**2012**	**S1**	38.30	*36.08-40.51*	2.80	*2.05-3.56*	17.80	*16.06-19.54*	4.26	*3.34-5.18*
	**S2**	34.93	*32.75-37.11*	1.69	*1.10-2.28*	9.86	*8.50-11.23*	5.67	*4.61-6.73*
	**S3**	39.43	*37.61-41.25*	3.08	*2.43-3.72*	15.86	*14.50-17.22*	*4.89*	*4.08-5.69*
	**S4**	35.90.	*35.14-36.67*	4.28	*3.96-4.60*	9.66	*9.19-10.14*	6.07	*5.69-6.45*

UI*. unspecified instrument.

### Group of public hospitals

Across the study period the proportion of caesareans performed in public hospitals ranged from 19.20% (CI 18.84-19.55) to 28.14% (CI 27.73-28.54). Comparison of the proportions for 2007 and 2012 by stratum shows that S3 hospitals present hardly any variations in the proportion of caesareans (*p =* 0.113). Among hospitals with the lowest annual numbers of births (S1 and S2) the proportion of caesareans decreased by 3.2% (*p =* 0.012) (S1) and 1.69% (*p =* 0.002) (S2) across the same period. By contrast, the proportion of caesareans performed increased significantly by 1.94% (*p =* 0.000) in hospitals with the highest annual numbers of births (S4).

The use of forceps showed a decreasing trend in public hospitals classified as S2 (p = 0.000), S3 (p = 0.000) and S4 (p = 0.000). Across both public and private hospitals the highest rate of forceps use in 2012 corresponded to public hospitals classified as S3 (8.98%; CI 9.24-8.72) and S4 (9.70%; CI 9.96-9.70).

The use of a vacuum extractor remained stable among S1 (*p =* 0,335) hospitals, but rose in S2 (*p =* 0.000), S3 (*p =* 0.000), and S4 (*p =* 0.000).

With regard to the use of non-specified instruments, proportions of this indicator decreased significantly in all four strata of public hospitals: S1 (*p =* 0.000), S2 (*p =* 0.000), S3 (*p =* 0.000), and S4 (*p =* 0.000). In 2012, the lowest proportion of vacuum use (1.15%; CI 1.06-1.25) corresponded to S4 hospitals, and the highest proportion (4.34%; CI 4.15-4.52) was found in S2.

### Group of private hospitals

The proportion of caesareans performed in private hospitals across the study period ranged from 32.21% (CI 31.78-32.63) to 39.43% (CI 38.98-39.87). Between 2007 and 2012 there was a 6.09% increase in the number of caesareans performed in hospitals classified in S1 (p = 0.000) and a 3.33% increase in the number carried out by S3 hospitals (p = 0.003). Over the same period the use of forceps declined across all four strata, most notably among S2 private hospitals.

The use of a vacuum extractor was more common among private hospitals, the highest rate corresponding to S1 hospitals. Comparison of the figures for 2007 and 2012 shows that the use of a vacuum increased notably over this period in S1 (p = 0.000) and S3 (p = 0.000) private hospitals.

The use of non-specified (spatula) instruments showed a clear decline between 2007 and 2012. The use of these instruments in the S3 hospitals fell by 3.21% (p = 0.000) and by 4.75% in S4 private hospitals (p = 0.000), but the decrease in S1 private hospitals was not significant (p = 0.023).

### Accredited hospitals

**Figure 3:**
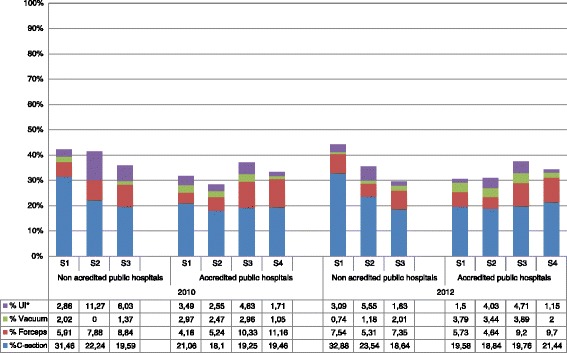
**Obstetric interventions in accredited and non-accredited public hospitals by stratum.**

Figure 3 shows data for the 44 public hospitals according to whether or not they were accredited to implement the normal childbirth initiative. In 2010 a total of 27 public hospitals had been accredited, with a further 5 achieving accreditation by 2012. The data are presented for each year and by strata (Figure 3). All the public hospitals classified as S4 (highest number of births/year) had been accredited by 2010.

The most common obstetric intervention performed in accredited hospitals was a caesarean. The overall proportions in this sub-group ranged from 18.10% (CI 17.15-19.06) to 21.06% (CI 18.19-23.93) in 2010 and from 18.84% (CI 17.91-19.77) to 21.44% (CI 20.74-22.14) in 2012.

No relevant differences in the obstetric interventions performed were observed between these two years in any of the strata. The use of forceps was more common in S3 and S4 accredited hospitals, and the greatest number of interventions in both years corresponded to S3 institutions.

### Non-accredited hospitals

In this sub-group the highest proportion of caesareans in both 2010 and 2012 corresponded to S1 and S2 hospitals. By summing the proportions corresponding to the columns in Figure 3 it can be seen that, in general, the four kinds of obstetric interventions considered in the present study are more commonly performed in non-accredited hospitals; note, however, that the proportion of interventions decreases progressively from S2 to S3 hospitals.

## Discussion

This paper forms part of a wider evaluation of maternity care services in Catalonia. The data used are derived from hospital discharge records that include diagnostic information and a description of any obstetric procedures used during labour. The paper focuses specifically on four obstetric interventions and examines changes in their use following implementation of the recommendations set out in a government strategy paper on normal childbirth. The indicators used here relate solely to interventions that may be performed during labour, a process which may also be influenced by other aspects of the maternity services available in a particular setting. In terms of the obstetric interventions that are performed, the findings reveal differences between public and private hospitals, and also between accredited and non-accredited public hospitals. This is especially evident with regard to caesareans, which have become more common in private hospitals over the study period considered here. This finding corroborates existing international previous research [15,4], as well as a study conducted in our geographical area [16]. It confirms the trend towards greater differentiation between public and private hospitals in this regard: the number of caesareans performed in public hospitals has remained stable in recent years, but in private hospitals it has risen.

The aim of this study was to provide a general overview of certain aspects of maternity services in Catalonia, both their organization (staffing) and some of the outcomes achieved. By grouping hospitals into different types and classifying them according to 1) the annual number of births recorded in their respective maternity service and 2) whether or not they are accredited to implement the normal childbirth initiative, it has been possible to observe differences that may be of key importance when it comes to further research and decision making in relation to healthcare policy.

In general, the number of obstetricians and midwives differs between public and private hospitals, and the employment situation of maternity health professionals and the institution also depends on the type of hospital. This could have implications for the kind of care they receive during labour with regard to the duration and type of care. This highlights the need to study other factors that may be relevant to the delivery of clinically and economically effective services [17,18]: for example, what sort of employment contract the staff should have, the kind of professionals who should be hired, the number of hours they need to work and the experience required by maternity care staff.

In the present study, hospitals were stratified according to the annual number of births recorded in their respective maternity service. The results showed that, in general, the highest numbers of obstetric interventions were performed by hospitals with a lower annual number of births. This could be interpreted as a negative finding, since in Catalonia hospitals are classified in three levels [19] according to their capacity to attend complications. According to this classification the hospitals where fewer births take place are also the ones that are less well equipped to deal with complicated births, and they tend to provide care to women at low obstetric risk. Research suggests that women at low obstetric risk are less likely to undergo an assisted birth in hospitals with smaller maternity departments or in ‘birth centres’ that operate a policy geared towards normal childbirth [17,20,21]. The above finding therefore suggests that the current model of maternity care in these Catalan hospitals needs to be reconsidered in light of the implications it may be having for outcomes.

Caesareans were performed more often in private than in public hospitals. There were also differences between public and private hospitals in the distribution of proportions for the other kinds of obstetric interventions considered here. Our findings are consistent with previous studies that have compared the maternity outcomes of public and private hospitals either for the population as a whole or among women at low obstetric risk [15,22]. Our analysis showed that the use of a vacuum extractor is now more common and appears to be on the rise in private hospitals. While the use of forceps has declined overall, this kind of assisted birth is still more frequent in public than in private hospitals. Numerous studies have concluded that differences in the kind of obstetric interventions performed may be attributable to the type of hospital (public or private), in that the interventions used are not always justifiable in terms of the obstetric risk presented [2,22]. These findings highlight the need to examine whether such practices have a negative impact on maternal or neonatal health.

A final result to consider from the analysis of public hospitals is that fewer caesareans were performed in hospitals accredited to implement the *Strategy for Assistance at Normal Childbirth* than in hospitals that were not accredited. This finding highlights the importance of continuing to promote the recommendations in this strategy in all hospitals [14].

When new health policies are implemented, their impact must be periodically evaluated. It is important to know the opinions of service users. Much of the data used by public administrations in this regard is derived from hospital discharge records, which can be used to establish quality indicators and to examine how practices (in this case, obstetric intervention) may have changed since a new policy was implemented [23,24]. If our aim, as policy makers, is to explore the extent to which maternity services have become more women-centred, then data of this kind cannot provide exhaustive information [25,26], although they do have a role to play provided they are complemented by information obtained from women themselves and from professionals [27,28]. Some studies have used medical records and interviews with women to gather more detailed information about the maternity care received, since on many occasions there will be information recorded in the medical notes that is not mentioned in the discharge report. This reinforces the recommendation to record all treatment or interventions in a patient’s medical records [29,3], and suggests the need for further consideration regarding the data that should be included in discharge reports.

This study aims to evaluate the impact that policymaking and national recommendations for normal childbirth care have on clinical practice. For this purpose, the hospital has been taken as the unit of analysis, obviating potentially different inter-professional practices.

We are aware that the characteristics of women attending private or public hospitals may vary and they could potentially affect the results.

This study did not consider clinical conditions, for example, whether caesarean sections were emergency or planned, since our objective was to analyses global intervention rates. The standards recommended in the *Strategy for Assistance at Normal Childbirth* on the different obstetric interventions discussed in this paper are assumed. These standards are useful as a reference to identify high intervention rates.

## Conclusions

Caesareans are the most common obstetric intervention performed in the context of full-term singleton births in Catalonia. The number of caesareans carried out in public hospitals has remained stable, whereas there is an upward trend in the use of this procedure by private hospitals. The use of a vacuum extractor has become more common, most notably among private hospitals.

In the sub-group of non-accredited public hospitals the highest proportion of caesareans corresponded to those hospitals with the lowest annual number of births (S1), and this proportion increased between 2010 and 2012. Among accredited public hospitals the proportion of caesareans was within a limited range in all four strata (i.e. regardless of the annual number of births they recorded), and it remained stable over the study period.

Analysis of staff profiles according to the stratification of hospitals by annual number of births showed that almost all the hospitals (with the exception of S3 public hospitals) had more obstetricians than midwives among their maternity care staff.
